# Gastrin releasing peptide receptor targeted nano-graphene oxide for near-infrared fluorescence imaging of oral squamous cell carcinoma

**DOI:** 10.1038/s41598-020-68203-y

**Published:** 2020-07-10

**Authors:** Ran Li, Ruifang Gao, Yimei Wang, Zhuanzhuan Liu, Hang Xu, Ailin Duan, Fang Zhang, Lixin Ma

**Affiliations:** 1 Shanxi Medical University School and Hospital of Stomatology, Taiyuan, 030001 China; 2 Research Division/Biomolecular Imaging Center, Harry S. Truman Memorial Veterans’ Hospital, Columbia, MO 65201 USA; 3 Department of Radiology, University of Missouri, Columbia, MO 65212 USA; 4 Present Address: School of Pharmacy, Nanjing Medical University, Nanjing, 211100 Jiangsu China

**Keywords:** Cancer imaging, Head and neck cancer, Oral cancer, Graphene, Nanobiotechnology, Nanomedicine

## Abstract

Oral squamous cell carcinoma (OSCC) is the most common malignant tumor that occurs in the oral mucosa. Pathological biopsy is still the current gold standard for OSCC diagnosis; however, some drawbacks need to be overcome. Therefore, it is urgently needed to find a non-invasive targeted technology for OSCC early diagnosis. Fluorescent optical imaging using near infrared (NIR) dyes tagged to tumor specific target will benefit such developments. Gastrin releasing peptide receptor (GRPR) is an attractive target for OSCC imaging and therapy. In this study, we synthesized nano-graphene oxide (NGO) nanoparticles with GRPR-specific peptides AF750-6Ahx-Sta-BBN via hydrogen bond and π–π bonds (NGO-BBN-AF750), and investigated their receptor binding, cell uptake and internalization in HSC-3 cells. NGO-BBN-AF750 and AF750-6Ahx-Sta-BBN showed a similar binding affinity to GRPR on HSC-3 cells. In contrast to AF750-6Ahx-Sta-BBN antagonist peptide, NGO-BBN-AF750 showed cellular internalization property. Overall, this study proposes a NGO nanoclusters-based nanoprobe for GRPR targeted near-infrared fluorescence imaging for OSCC. Nanoparticle-based delivery systems have shown highly significant potential in the delivery of a wide range of therapeutic agents.

## Introduction

Oral squamous cell carcinoma (OSCC) is the most common malignant tumor that occurs in the head and neck^1^. Despite many advanced therapies, the 5-year survival rate of OSCC patients still stagnate at 40–50%. Because of lack of effective diagnostic approaches, over 60% of patients present stages III and IV at the time of diagnosis^2^. The histopathological examination of suspected oral mucosa biopsy tissues is considered a gold standard for validation of OSCC^3^. However, it has limitations such as laborious, causing pain and time-consuming. In addition, patients with any form of suspected lesion may need to undergo a second biopsy for further confirmation. Therefore, developing sensitive screening methods that are non-invasive and economic, would be necessary to enhance early diagnosis of OSCC and improve the patients’ survival. And, effective screening aids to differentiate benign from malignant lesions as well as to avoid complications associated with false diagnosis of oral cancer. Recently, it is proven that optical imaging systems are effective for cancer imaging in hollow organs or as intraoperative imaging tools^4,5^.

Gastrin-releasing peptide receptor (GRPR) is an attractive target for OSCC imaging and therapy. GRPR, a G protein-coupled receptor, has been proven with high expressions on many human tumors, such as prostate cancer, gastrointestinal stromal, breast cancer, ovarian cancer and small cell lung cancer^6–12^. Recently, Lango MN^13^ found that GRPR is overexpressed in both head and neck squamous cell carcinoma (HNSCC) tumors and adjacent normal mucosa from HNSCC patients compared with levels in control mucosa from individuals without cancer. Other researchers have also shown that other tumors overexpress GRPR on their cell surface, including head/neck^14^. GRPR was further used as a biomarker for surgical margin prediction in a murine orthotopic model of oral cancer and a strong expression of GRPR was observed uniformly in primary OSCC sections as compared to respective adjacent non-malignant region^15^. Bombesin (BBN) peptide has shown high binding affinity and specificity to target the GRPR; efforts have been focused on developing radiolabeled or fluorescent dye labeled BBN analogues for tumor imaging and therapy^14,16–19^. BBN peptides, both agonist and antagonist, can be efficiently conjugated to various ligands such as metal chelators, near infrared fluorescence (NIRF) dyes, and nanoparticles^7,20,21^.

In the past decades, great progress has been made in the field of nanotechnology. Nanoparticles are widely used in various biomedical applications, including imaging, diagnosis and therapeutic agents^22–25^, due to their small size, easy surface modification and effective binding with biomolecules. Nano-graphene oxide (NGO) has a characteristic of large surface area, good water dispersibility and biocompatibility, facile surface modification, and low manufacturing cost, making it a promising candidate for biological applications^26^.

Herein, we develop a GRPR targeted molecular imaging method and investigate the feasibility of its application in early diagnosis of OSCC. In this study, we used a GRPR-specific Alexa Fluor 750 (AF750) labeled BBN antagonist, AF750-6Ahx-Sta-BBN, to evaluate the expression of GRPR in OSCC and normal epithelial tissues by immunofluorescence, and the binding selectivity and affinity to human oral cancer cell line HSC-3 cells in vitro. We further synthesized a NGO-BBN-AF750 nanoparticle by combining NGO with AF750-6Ahx-Sta-BBN peptides via hydrogen bond and π–π bonds, and investigated their receptor binding, cell uptake and internalization in HSC-3 cells.

## Results

### Immunofluorescence assay shows high GRPR expressions in OSCC tissues

**Figure 1:**
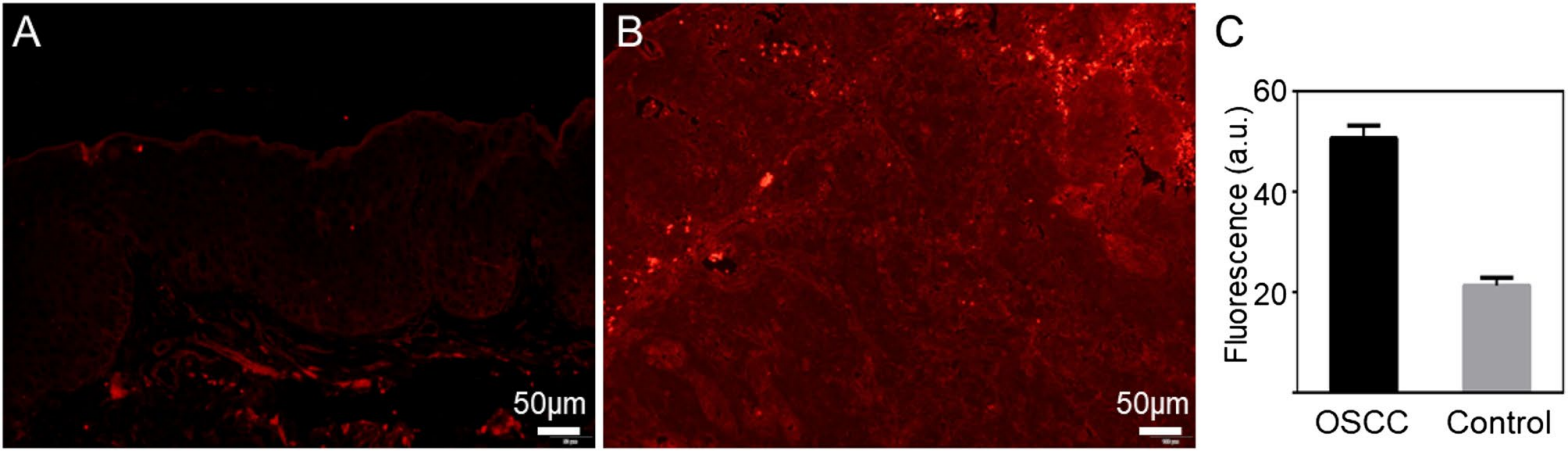
Immunofluorescence assay of paraffin sections from (**A**) normal oral mucous tissue and (**B**) OSCC tissue. (**C**) Quantitation of GRPR expression.

We analyzed the expression levels of GRPR in primary OSCC sections and the normal tissue sections using AF750-6Ahx-Sta-BBN immunofluorescence assay. The OSCC tissues showed a strong fluorescence signal and a 2.5-fold intensity increases compared with the normal oral mucous tissue (Fig. 1).

### Synthesis and characterization of NGO-BBN-AF750

**Scheme 1:**
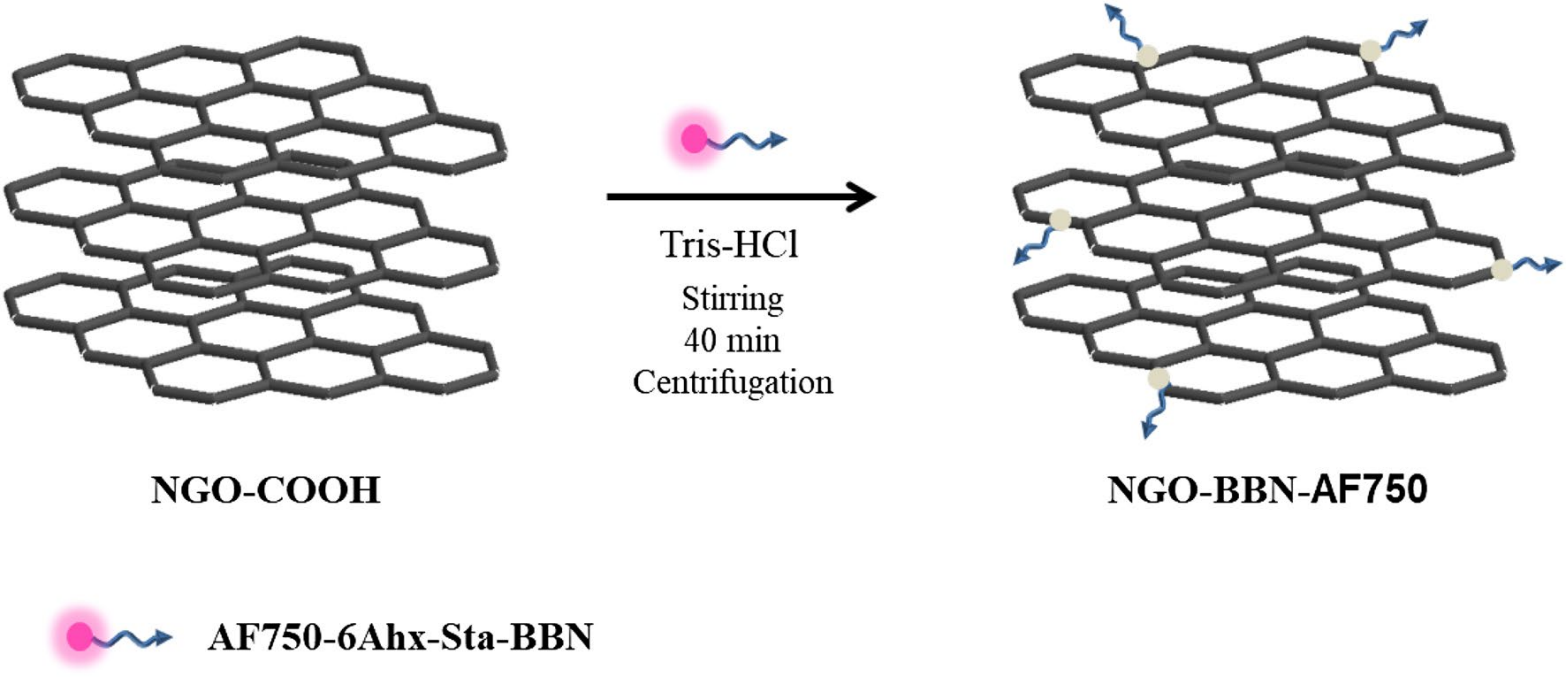
Schematic of fabrication of NGO-BBN-AF750, nano-graphene oxide equipped with bombesin peptides via hydrogen bond and π–π bonds.

**Figure 2:**
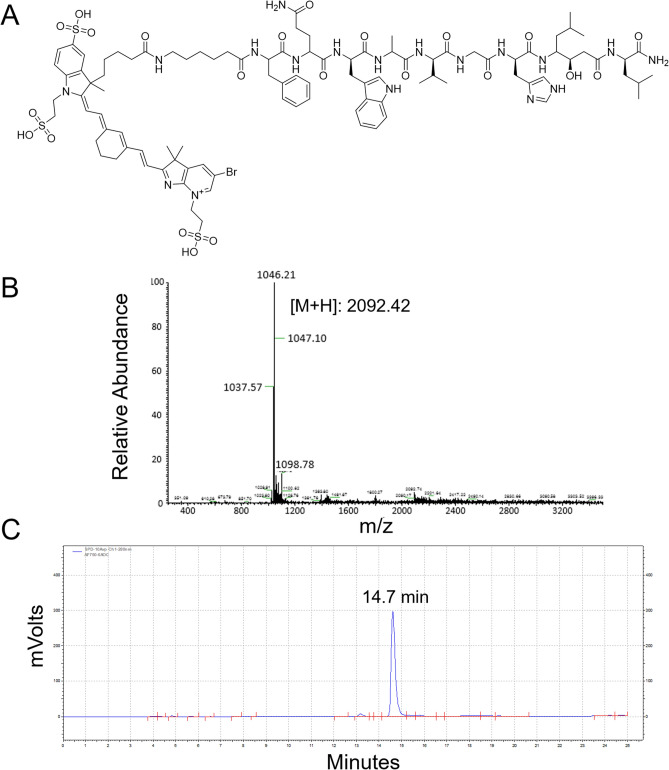
(**A**) Chemical structure of AF750-6Ahx-Sta-BBN, and its (**B**) mass spectrum (expected [M + H]: 2,092.914) and (**C**) RP-HPLC profile (retention time: 14.7 min. HPLC gradient: 0–15 min: 20–40%, 15–20 min: 40–80%, 20–25 min: 80–20%; flow rate 1 mL/min; buffer a: 0.1% TFA in H_2_O, buffer b: 0.1% TFA in acetonitrile, on a Phenomenex 5 µm C18 300 Å 250 × 4.6 mm column).

**Figure 3:**
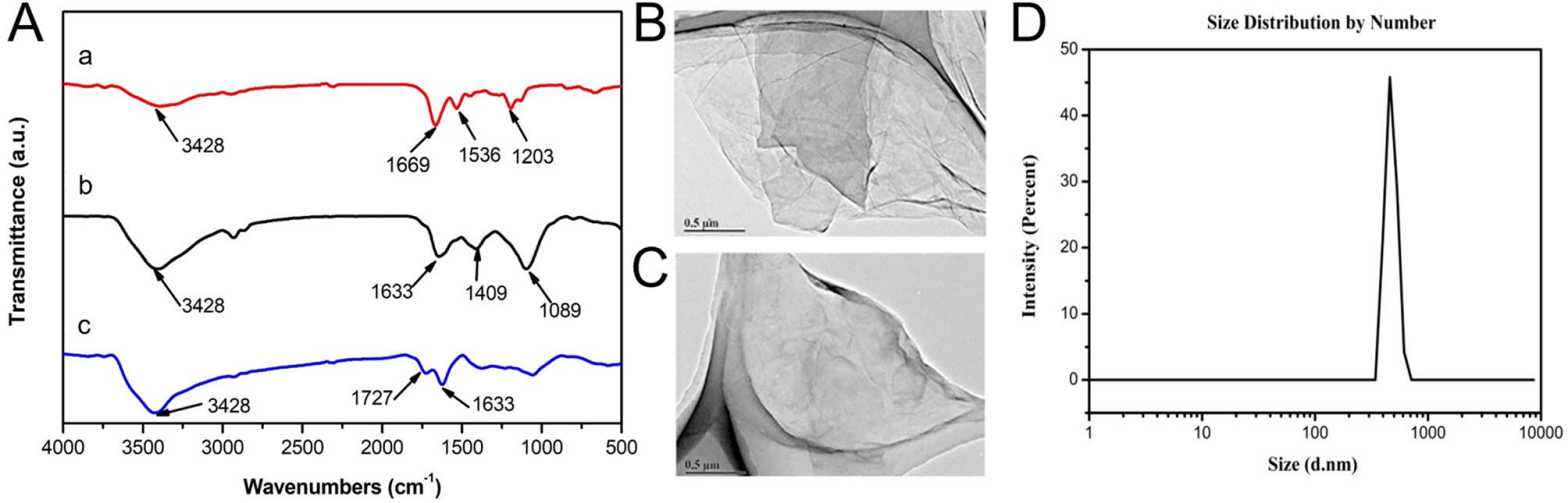
(**A**) Fourier-transform infrared spectroscopy of AF750-6Ahx-Sta-BBN (a), NGO-COOH (b) and NGO-BBN-AF750 (c). TEM of (**B**) NGO-COOH and (**C**) NGO-BBN-AF750. (**D**) Hydrodynamic diameter distribution of NGO-BBN-AF750 determined using dynamic light scattering (DLS).

NGO-BBN-AF750 was synthesized by coupling the NGO-COOH with AF750-6Ahx-Sta-BBN via hydrogen bond and π–π bond, and purified with centrifugation (Scheme 1). AF750-6Ahx-Sta-BBN was synthesized and purified (Fig. 2) as described previously^27^. NGO-BBN-AF750 showed a zeta potential of − 16.56 Mv. FT-IR spectra of NGO-COOH, NGO-BBN-AF750 and AF750-6Ahx-Sta-BBN, the TEM of NGO-COOH and NGO-BBN-AF750, and the hydrodynamic diameter profile of NGO-BBN-AF750 are shown in Fig. 3. For NGO-COOH, the FT-IR characteristic peak appeared at 3,428 cm^−1^. The band at 1,727 cm^−1^ was owing to symmetric stretching of –COOH, whereas vibration peak at 1,633 cm^−1^ was due to adsorbed water molecule. For AF750-6Ahx-Sta-BBN peptide, the infrared peak at 3,428 cm^−1^ was the symmetric stretching of –OH. The band at 1,669 cm^−1^ represented the vibration of C=O. The peaks at 1,536–1,203 cm^−1^ were due to benzene rings and other functional groups. For NGO-BBN-AF750, the peak for –COOH of NGO disappeared, and a new peak was observed at 1,409 cm^−1^. The changes in these chemical bond vibration peaks confirmed the bond formations of NGO with AF750-6Ahx-Sta-BBN peptides, through hydrogen bond and π–π bond interactions.

**Figure 4:**
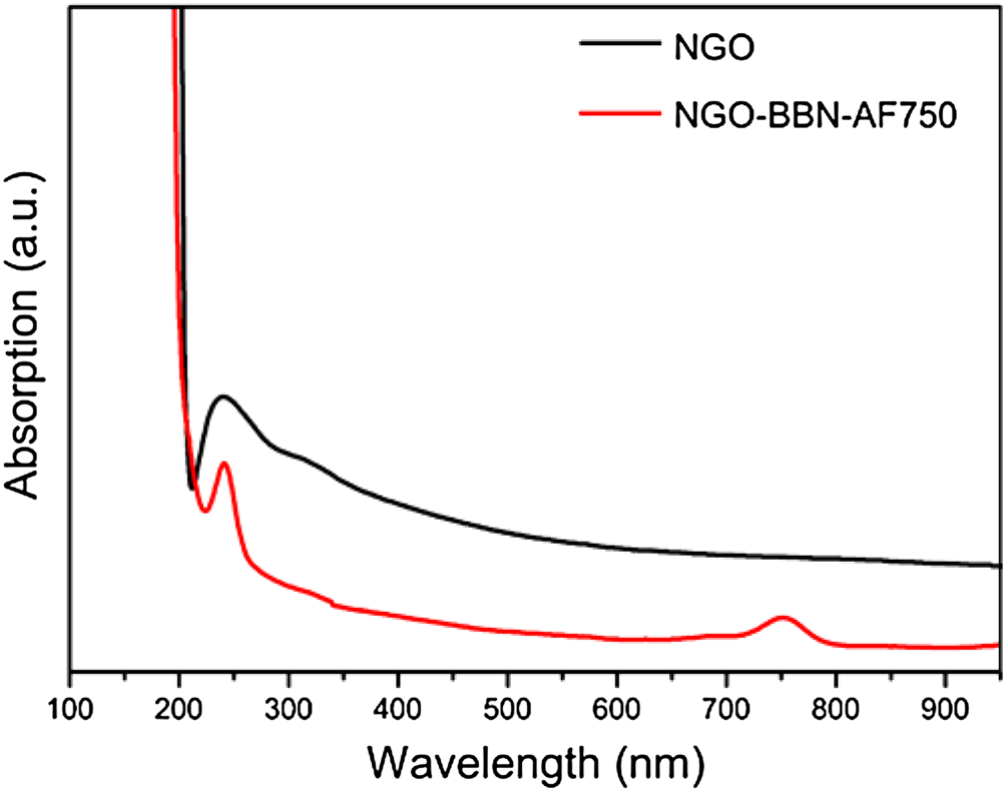
UV–Vis spectra of NGO and NGO-BBN-AF750 nanoprobes.

Furthermore, the UV–Vis spectra of NGO and NGO-BBN-AF750 showed that NGO-BBN-AF750 had a strong absorption at 750 nm and a disappearance of the broadband peak at 320 nM compared with NGO (Fig. 4). Both the appearance of the peak at the NIR wavelength 750 nm and the disappearance of the broadband absorption at 320 nm further indicate the surface modification of the NGO with the AF750-6Ahx-Sta-BBN peptides.

### Fluorescence quenching and restoration

**Figure 5:**
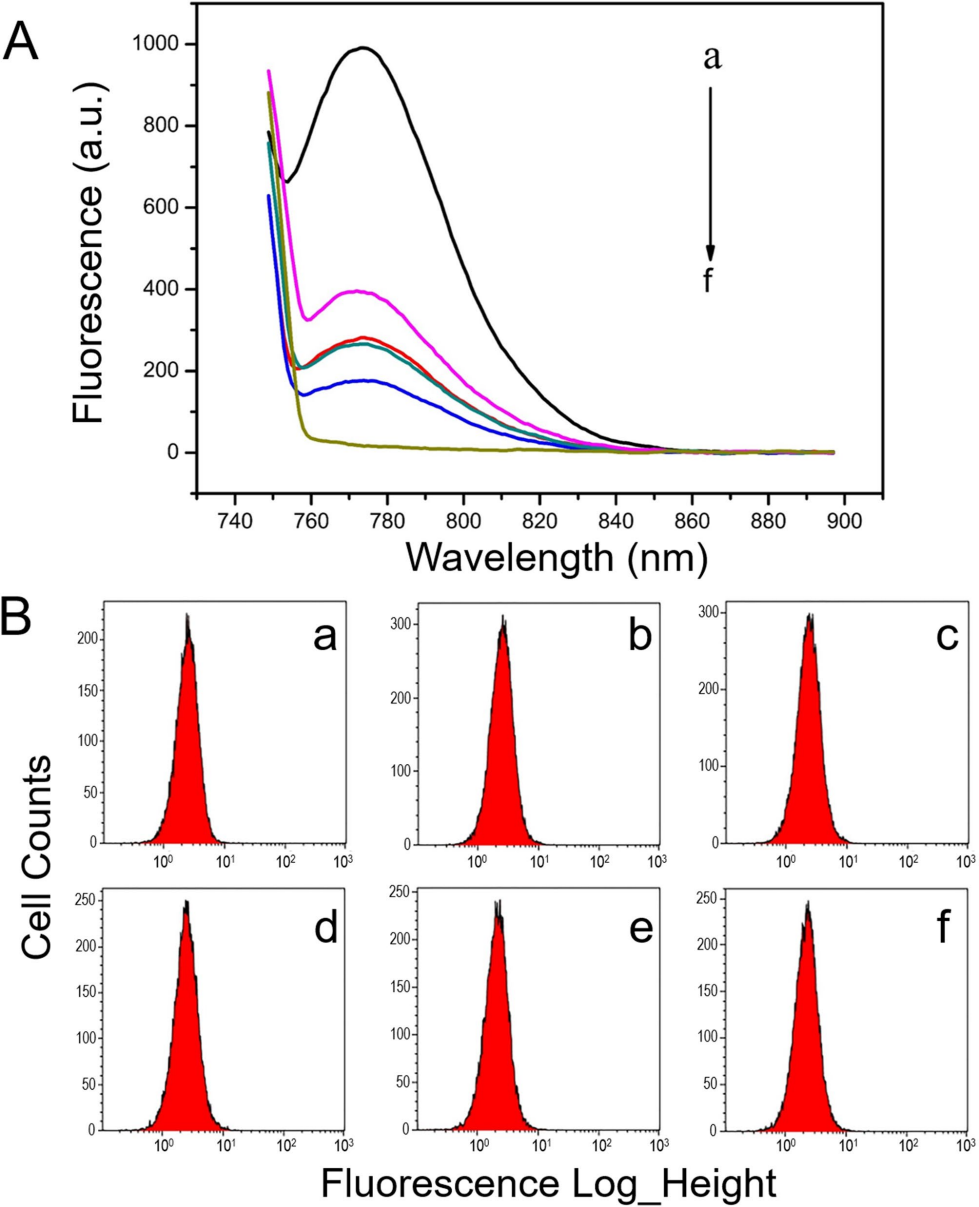
Fluorescence quenching effect of NGO. (**A**) The NGO extinction efficacy of fluorescence was measured with fluorescence spectrophotometer. The fluorescence intensity of AF750-6Ahx-Sta-BBN decreases with increase of concentrations of NGO (a–f: for 0, 0.01, 0.02, 0.04, 0.08, 0.1 mg/mL, respectively). (**B**) Fluorescence recovery of the probes formed by NGO after incubation with HSC-3 for 40 min; (a–f) histogram of HSC-3 cell counts for the probes at the concentration of NGO: 0, 0.01, 0.02, 0.04, 0.08, 0.1 mg/mL, respectively.

It has been reported that NGO has fluorescence extinction effect. In order to explore the extinction efficiency of NGO to AF750 fluorescent probes, different concentrations of NGO were prepared into six groups: 0, 0.01, 0.02, 0.04, 0.08 and 0.1 mg/mL, and each mixed with 50 nM AF750-6Ahx-Sta-BBN in buffer solutions. The extinction efficacy of fluorescence was measured with fluorescence spectrophotometer. As shown in Fig. 5A, the AF750 fluorescence peak intensity at approximately 772 nm decreases with increase of NGO concentrations. When the concentration of NGO was 0.1 mg/mL, the AF750 fluorescence intensity was completely eliminated.

However, the quenched fluorescence intensity of the probes was recovered after incubation with 2 × 10^6^ HSC-3 cells for 40 min and detected with flow cytometry. As Fig. 5B shows, equal amount of fluorescent probe targeted HSC-3 cells were detected in all six groups, despite the strong fluorescence intensity quenching effect at 0.1 mg/mL NGO (Fig. 5B). Thus, 0.1 mg/mL NGO was selected for the in vitro analytical purposes. The results indicated that BBN-AF750 could shed from the probe and play a targeting role when the probe encounters GRPR.

### Cell binding experiment

**Figure 6:**
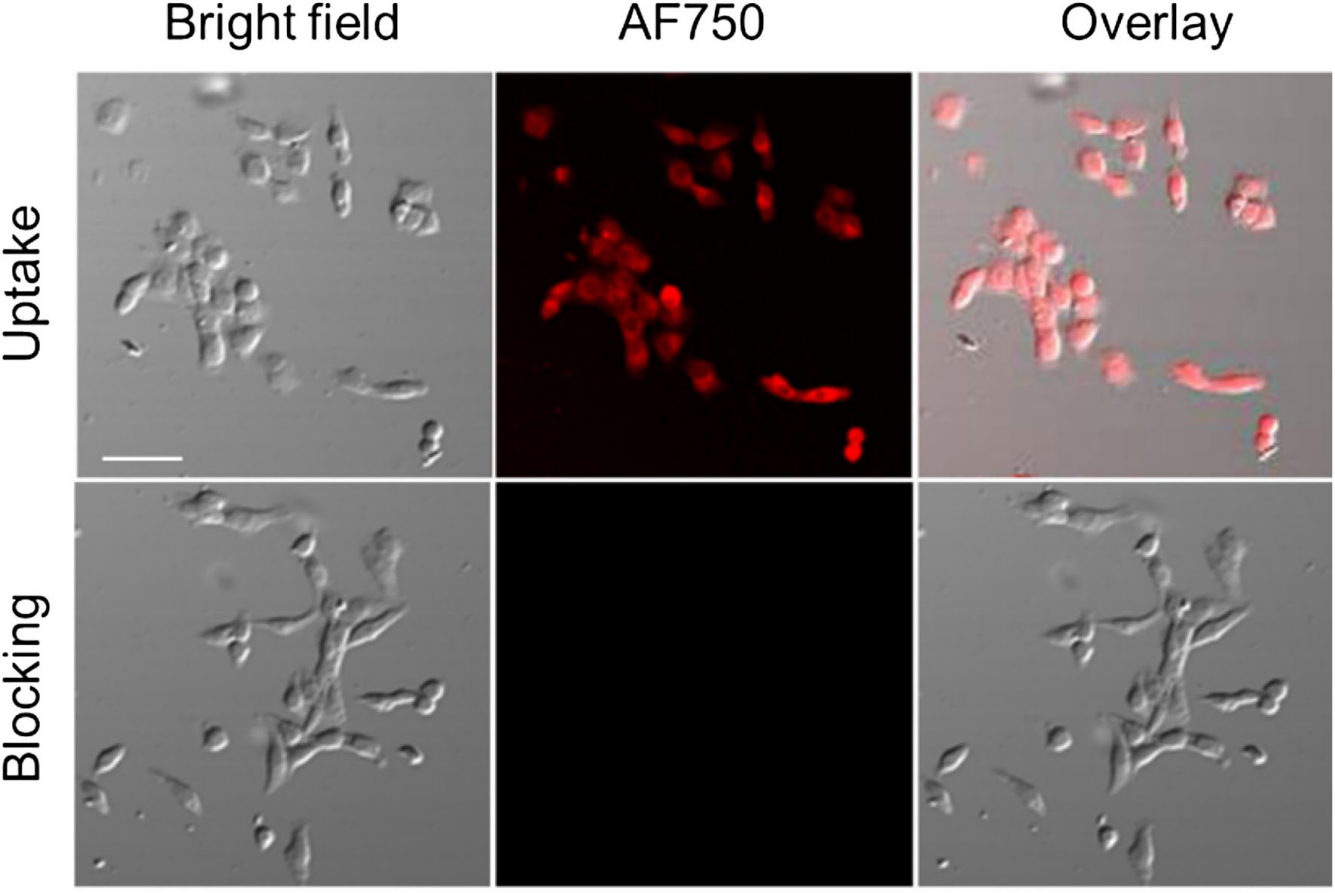
The specificity binding assay of AF750-6Ahx-Sta-BBN to GRPR on HSC-3 cells at 37 °C. The scale bar is 30 μm.

**Figure 7:**
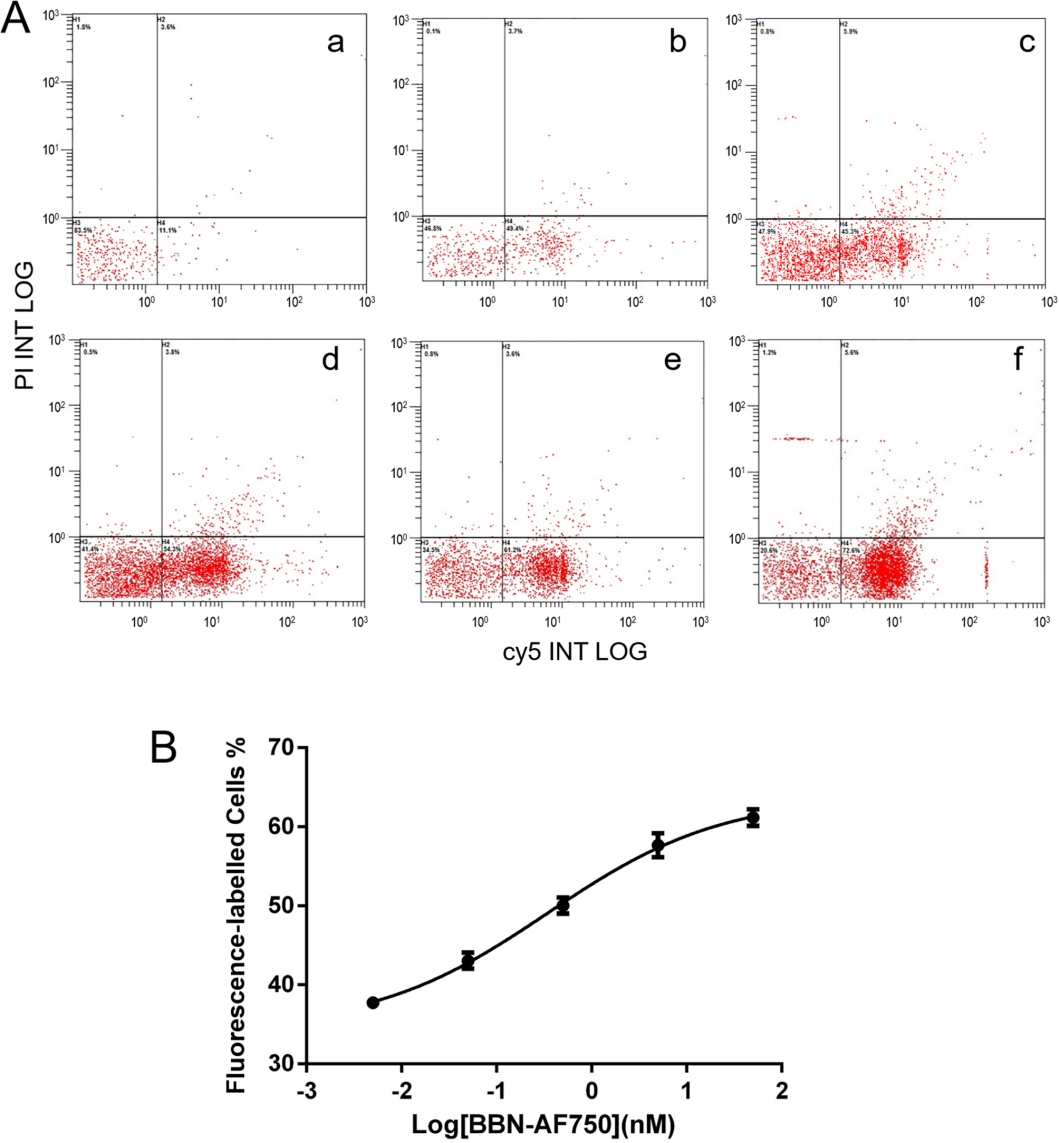
In vitro competitive GRPR binding assay in HSC-3 cells. (**A**) Histograms of fluorescent labelled HSC-3 cells at different concentrations of AF750-6Ahx-Sta-BBN (a–f: 0, 0.005, 0.05, 0.5, 5, 50 nM). (**B**) The EC50 of AF750-6Ahx-Sta-BBN was estimated as 0.47 ± 1.05 nM for HSC-3 cells.

The binding specificities of AF750-6Ahx-Sta-BBN were determined with HSC-3 cells of human oral tongue squamous cell carcinoma using previously described methods^27^. As shown in Fig. 6, the HSC-3 cells in the uptake group where cells were incubated with AF750-6Ahx-Sta-BBN displayed strong NIR fluorescence signals, while the HSC-3 cells in the blocking groups where cells were incubated with excessive BBN (1–14) peptide for 10 min prior to incubation with AF750-6Ahx-Sta-BBN had no fluorescence signal. The result indicates that AF750-6Ahx-Sta-BBN specifically binds to the GRPR on HSC-3 cells. To determine the binding affinity of AF750-6Ahx-Sta-BBN in HSC-3, cells were first incubated with BBN (1–14), then added increasing concentrations of AF750-6Ahx-Sta-BBN (0.005–50 nM). Cells were washed after 50 min incubation and examined by flow cytometry. The binding affinity to GRPR is shown in Fig. 7, and plotted as the fluorescence intensity as function of the AF750-6Ahx-Sta-BBN concentration (Fig. 7B). The half maximal replacement concentration (EC50) value of AF750-6Ahx-Sta-BBN was 0.47 ± 1.05 nM, the result further confirmed the specific interaction of AF750-6Ahx-Sta-BBN to the GRPR on HSC-3 cells.

**Figure 8:**
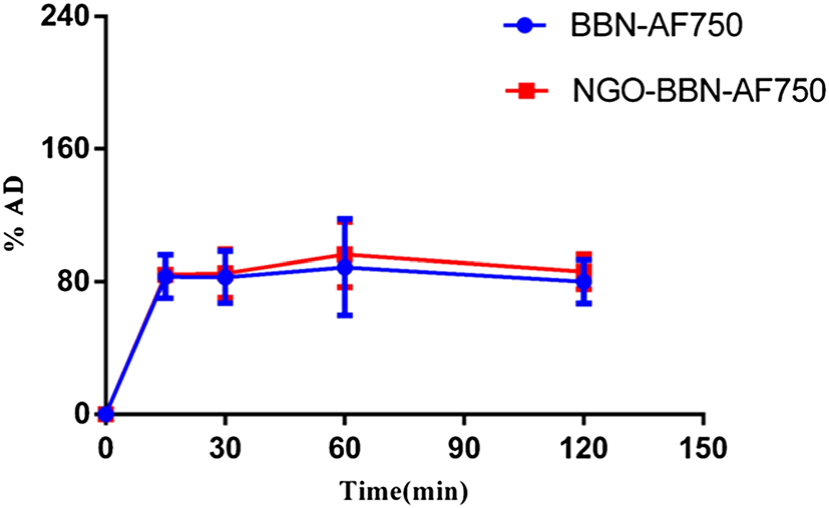
In vitro cell binding studies of AF750-6Ahx-Sta-BBN and NGO-BBN-AF750 at different time at 37 °C.

**Figure 9:**
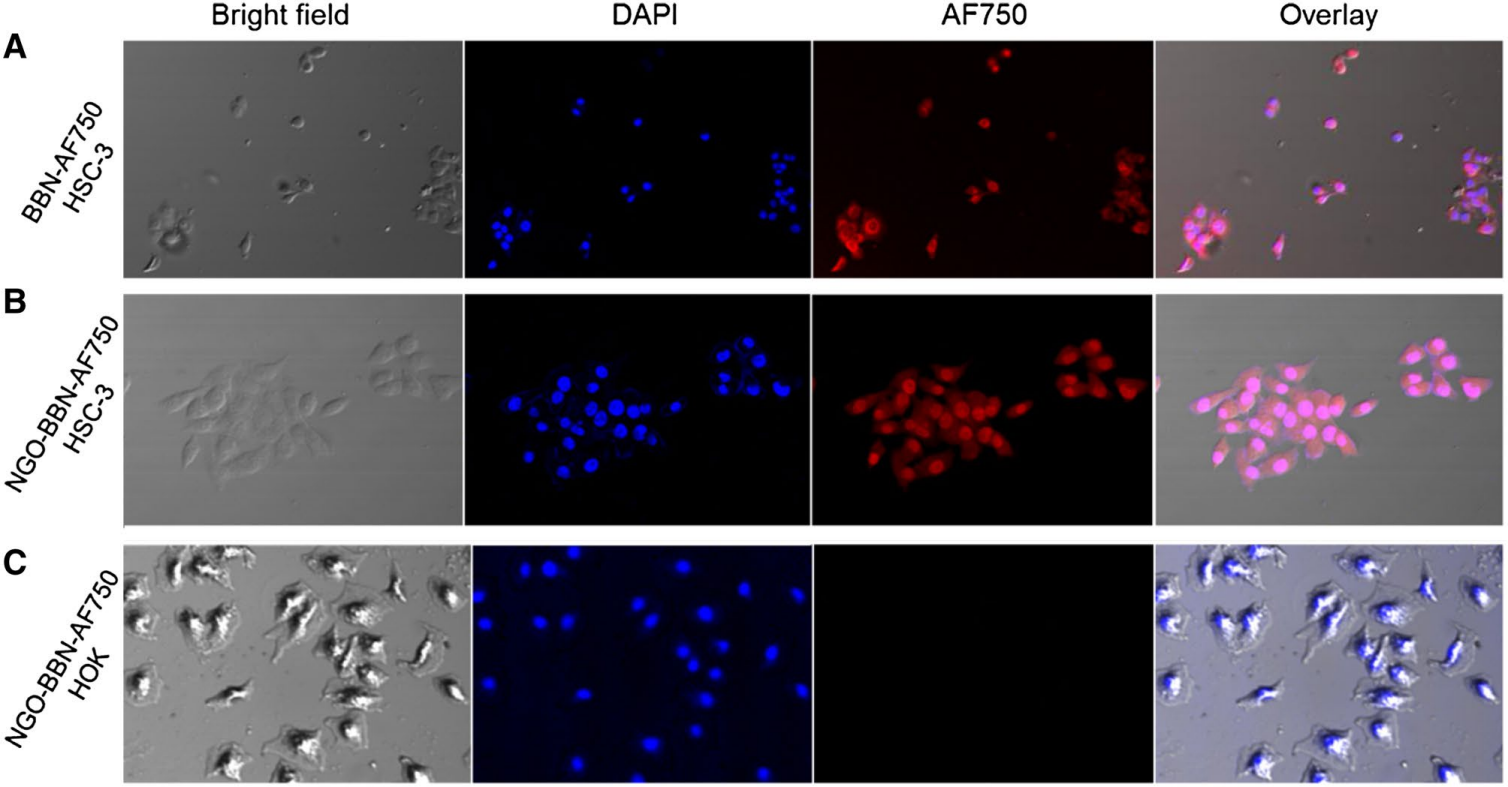
Microscopic images show cell binding of (**A**) AF750-6Ahx-Sta-BBN and (**B**) NGO-BBN-AF750 on HSC-3 cells. (**C**) NGO-BBN-AF750 does not bind to the GRPR-negative HOK cells.

**Figure 10:**
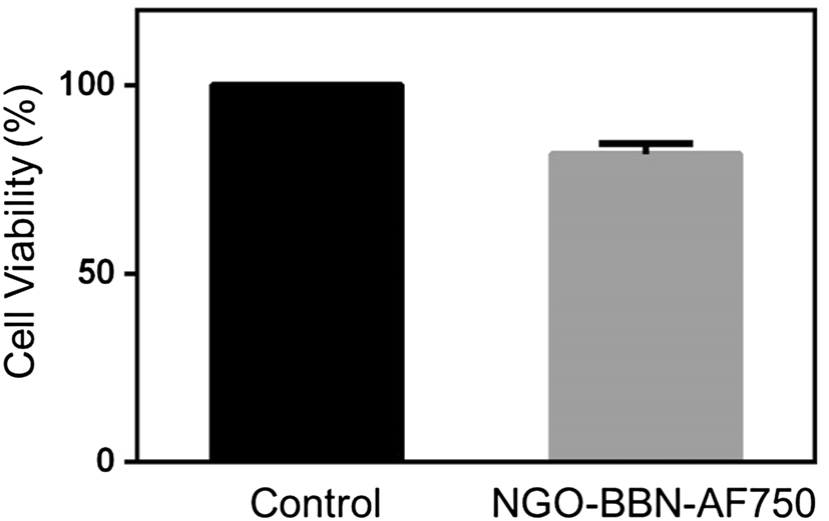
Cell viability treated without (control) and with NGO-BBN-AF750.

The time-dependent experiment was carried out on HSC-3 cells for both AF750-6Ahx-Sta-BBN and NGO-BBN-AF750. The results showed the time-dependent uptake of the probes by HSC-3 (Fig. 8). With increase of incubation time, cells uptake increased, and at the first 15 min, we observed a rapid binding of up to 80%. Whereas, after 15 min, the fluorescence showed little increase with time. The cell uptake of NGO-BBN-AF750 was not significantly different compared with BBN-AF750, indicating that NGO had no adverse effect on the BBN binding affinity. The cell uptake was 83 ± 15% and 89 ± 29% (AF750-6Ahx-Sta-BBN) and 85 ± 15% and 96 ± 20% (NGO-BBN-AF750) at 30 min and 60 min, respectively. We further conducted confocal laser scanning microscope experiments to evaluate the internalization of AF750-6Ahx-Sta-BBN and NGO-BBN-AF750 into HSC-3 cells and HOK cells. Blue fluorescence (Excitation, 460 nm) was used to represent the nuclei of HSC-3 cells. Red (Excitation, 750 nm) channel represents the fluorescence signal of AF750. As shown in Fig. 9, AF750-6Ahx-Sta-BBN was observed on the membrane of HSC-3, agreeing with its GRPR antagonist’s property on cell surface^27^. However, stronger fluorescence signals were appeared overlapping with the nucleus of HSC-3 cells in the NGO-BBN-AF750 group, indicating a cell internalization activity by NGO-BBN-AF750. No fluorescence was observed in HOK cell which was selected as a negative control as they do not express GRPR. Taken together, both AF750-6Ahx-Sta-BBN and NGO-BBN-AF750 showed a high specificity to HSC-3 cells mediated by the GRPR binding. While AF750-6Ahx-Sta-BBN is a GRPR antagonist to the HSC-3, NGO-BBN-AF750 may be internalized into HSC-3 cells via the combined interaction of BBN binding and the endocytosis of the nanoparticle. Evaluating nanotoxicity of NGO-BBN-AF750 was a part of our experimental design. Preliminary in vitro experiments showed that NGO-BBN-AF750 had a decrease in cell viability (82 ± 2%) compared to the control (Fig. 10).

## Discussion

OSCC is a common type of head and neck squamous cell carcinoma^28^. Although pathological biopsy is still the current gold standard for OSCC diagnosis, due to its invasiveness and needs for repeated sampling at the same site^29^, it is urgently needed to find a non-invasive and targeted technology for OSCC early detection. Actively targeting tumors with cancer-specific molecules containing fluorescent groups plays an important role in the diagnosis and treatment of OSCC. In recent years, GRPR has been used as a target for cancer diagnosis and treatment of prostate cancer, breast cancer and small cell lung cancer^30–32^. Few studies were conducted to investigate the GRPR targeting potential in human head and neck squamous cell carcinoma OSCC. Recently, Lango et al.^13^ had shown that GRPR expression in head and neck squamous cell carcinoma is six times higher than that in non-cancer tissues, four times higher than that in normal epithelial tissues adjacent to cancer tissues. GRPR is highly expressed in early stage of OSCC. In the present study, we also demonstrated the high expression level of GRPR in primary OSCC tissues by immunofluorescence, and in the human tongue squamous cell carcinoma cell line HSC-3. Our analyses showed that GRPR expression in OSCC tissue was 2.5 times higher than that in normal oral mucosa tissue, which is slightly lower compared with the study by Lango et al.^13^. The possible reason may be that the oral squamous cell carcinoma tissue selected was highly differentiated tissue in our study.

AF750-6Ahx-Sta-BBN had previously been shown with a high binding affinity and specificity to the GRPR overexpressed in a prostate cancer PC-3 cell line^27,33^. In the present study, we showed that AF750-6Ahx-Sta-BBN has a high binding affinity and specificity to the human tongue squamous cell carcinoma cell line HSC-3, and also to the primary OSCC tissue sections. AF750-6Ahx-Sta-BBN is a GRPR antagonist peptide analog. Siyuan Cheng et al.^34^ compared GRPR agonist BBN (7–14) and GRPR antagonist RM26 for prostate cancer PET imaging and found that GRPR antagonist is a candidate for clinical transformation. This present study is the first study to demonstrate the potential of using a GRPR antagonist AF750-6Ahx-Sta-BBN as a potential near-infrared fluorescence imaging probe for OSCC detection.

Compared with pristine graphene, NGO contains many oxygen-containing functional groups (including hydroxyl, carboxyl and oxygen-containing groups), and has a large surface area and good optical absorption in the near infrared region^35–37^. At the same time, NGO has EPR effect. Therefore, it attracts attention to study as tools for the diagnosis and treatment of cancer. NGO interacts dynamically with ligand probes through fluorescence, Raman scattering and electrochemical reactions, and/or has been used to transduce specific responses to target molecules^37^. In fact, graphene and NGO have been functionalized with nucleic acids, peptides, proteins, aptamers, small molecules, bacteria, and even cells through physical adsorption or chemical conjugation^38,39^. Meanwhile, it is said that graphene and NGO is a general quencher, and a diverse range of species (such as fluorophores, quantum dots, and fluorescent metal nanoclusters) can be efficiently quenched^39,40^. Our previous research investigated the detection of promyelocytic leukemia/retinoic acid receptor α(PML/RARα) fusion gene with functionalized graphene oxide and fluorescence probe analysis of leukemia cells by modified graphene oxide^41^. In this study, NGO carboxylation was used to increase its water solubility and stability, and then NGO was coupled with AF750-6Ahx-Sta-BBN via hydrogen bond and π–π bond. Ultraviolet full wavelength scanning and Fourier infrared spectroscopy showed that NGO was successfully coupled with AF750-6Ahx-Sta-BBN. We studied the fluorescence quenching of BBN-AF750 with different concentrations of NGO. When the concentration of NGO was 0.1 mg/mL, the maximum fluorescence quenching was achieved, and the fluorescence was restored in HSC-3 cells upon the interaction between NGO-BBN-AF750 and HSC-3 cells.

In this study, we evaluated the uptake and internalization of NGO-BBN-AF750 and AF750-6Ahx-Sta-BBN in cells. Flow cytometric analysis showed no statistical significance of tumor cell uptake for NGO-BBN-AF750 and AF750-6Ahx-Sta-BBN, indicating little adverse effect of NGO on the BBN binding to GRPR on HSC-3 cells. In contrast to AF750-6Ahx-Sta-BBN, NGO-BBN-AF750 showed a strong fluorescence signal overlapping with the nucleus, indicating that NGO-BBN-AF750 could be internalized into HSC-3 cells. Negative control HOK cells did not show any fluorescence. Internalization of NGO-BBN-AF750 could be explained by an added endocytosis interaction of NGO-BBN-AF750 with the cell membrane upon the active BBN binding to the cell surface GRPR. Study on long-term stability under complex biological conditions was not conducted in the current work, and will be subjected to future research.

## Conclusions

A gastrin releasing peptide receptor specific NGO nanoprobe was successfully prepared for OSCC NIR fluorescence imaging. The nanoprobe showed a high binding affinity and specificity to GRPR positive HSC-3 cells. Interestingly, compared with the AF750-6Ahx-Sta-BBN antagonist, NGO-BBN-AF750 could be internalized into HSC-3 cells through a combined interaction of BBN peptide mediated binding and the endocytosis of the nanoparticle. Our work provides a new idea for the diagnosis of oral cancer, and also offers a theoretical basis for OSCC therapy. Nanoparticle-based delivery systems have shown highly significant potential in the delivery of a wide range of therapeutic agents.

## Materials and methods

### Chemistry

Carboxylated nano-graphene oxide (NGO-COOH) was obtained from Nanjing XianFeng Nano Material Tech Co, Ltd. NaCl, KCl, MgCl and HCl were purchased from Sigma-Aldrich (Japan). Alexa Fluor 750 NHS ester was purchased from ThermoFisher Scientific (Waltham, MA, USA). NaHCO_3_, DMF and other regular reagents were purchased from Sigma-Aldrich (St. Louis, MO, USA). Full length bombesin[1–14] from American Peptide Company (Sunnyvale, CA, USA) was used as the blocking agent in this study. AF750-6Ahx-Sta-BBN was synthesized and purified at the Biomolecular Imaging Center, Harry S. Truman Memorial Veterans’ Hospital, and University of Missouri, Columbia, Missouri, United States, according to our published procedure^27^. Briefly, the N-terminus of the BBN antagonist peptide Sta-BBN (-DPhe-Gln-Trp-Ala-Val-Gly-His-Sta-Leu-NH_2_) was conjugated to the AF750 NHS ester (excitation/emission: 749/775 nm) via a hydrophobic linker 6-aminohexanoic acid (6Ahx, –NH–(CH_2_)5COOH–). The purity of the compound was determined > 95% using the RP-HPLC on an SCL-10A HPLC system (Shimadzu Corp., Kyoto, Japan). Mass spectrometry (MS) analyses were performed on a 4700 MALDI TOF/TOF mass spectrometer (Applied Biosystem Inc., now AB Sciex) at the University of Missouri Charles W. Gehrke Proteomics Center. Molecule weight (MW) of AF750-6Ahx-Sta-BBN was 2,092.4 in agreement with the expected 2,092.914 (Fig. 2).

### Synthesis of NGO-BBN-AF750 nanoprobes

Specifically, Tris–HCl was used as buffer, then 200 µL (0.1 mg/mL) NGO-COOH and 50 nM of AF750-6Ahx-Sta-BBN were added, stirred for 40 min in the dark, centrifuged, and the supernatant was removed to obtain NGO-BBN-AF750.

### Characterization

The surface functional groups of NGO-COOH and NGO-BBN-AF750 were analyzed with Fourier-transform infrared spectroscopy (Tensor-27, Bruker, Germany). The ultraviolet absorption spectra of NGO and NGO-BBN-AF750 were analyzed with ultraviolet–visible spectrophotometer (UV-3600, Shimadzu, Japan). The data analyses were performed using OriginPro 8. TEM was performed on JEOL JEM-2100F (JEOL Ltd., Japan). The hydrodynamic diameter profile of NGO-BBN-AF750 was determined with dynamic light scattering (DLS) using Zetasizer Nano ZS90 (Malvern Panalytical Ltd, UK). Samples were prepared by dissolving nanoparticles in deionized water. Their zeta potentials were also measured using Nano ZS90 (Malvern Panalytical Ltd, UK).

### Immunofluorescence assay

The study was approved by Ethics Committee of Shanxi Medical University (2019LL185) and all the experimental protocol and the methods were carried out in accordance with the relevant guidelines and regulations, and complied with the principles of the Declaration of Helsinki. Written informed consent signed by all participants.

The paraffin-embedded samples, obtained from the Department of Pathology, Stomatology Hospital of Shanxi Medical University, were cut into 4-µm slices. Then tissue blocks were treated with xylene and dehydrated using a graded ethanol series. Following that, the sections were heated for antigen retrieval and incubated in 3% H_2_O_2_ to block endogenous peroxidase. The BSA was added and incubated at 37 °C for 30 min. After that, BSA was removed and AF750-6Ahx-Sta-BBN was added and incubated for 10 min in the dark. The slides were observed and imaged using an inverted Olympus fluorescence microscope (Olympus Corp., Osaka, Japan). Quantitative analysis of GRPR expression was performed using ImageJ (https://imagej.nih.gov/ij/). Each patient received written informed consent before the study.

### Cell lines and cell culture

The human tongue squamous cell carcinoma cells (HSC-3) and human oral keratinocyte (HOK), purchased from the American Type Culture Collection (ATCC, Rockville, MD), were cultured in medium containing 10% fetal bovine serum (FBS; GIBCO) and 1% penicillin–streptomycin (GIBCO). Cells were cultured at 37 °C in a humidified atmosphere containing 5% CO_2_. Trypsin containing 0.25% EDTA was used to suspended cells. Cells were passaged in three days.

### In vitro cell binding affinity

In vitro quantification of binding affinity to GRPR was performed with the effective concentration 50 percent (EC50) assay. Briefly, a series of samples each with 1 × 10^6^ HSC-3 human OSCC cells in culture medium were incubated with 1.5 nM BBN(1–14), and added with increasing concentrations (5 × 10^–3^ nM to 5 × 10^1^ nM) of AF750-6Ahx-Sta-BBN or NGO-BBN-AF750 at 37 °C and 5% CO_2_ for 50 min. The incubation medium was subsequently aspirated, and the cells were washed three times with ice cold media. Fluorescence labeled cells was analyzed using flow cytometry with Beckman Coulter Navios flow cytometer (Beckman Coulter Life Sciences, USA). Experimental data were processed using GraphPad Prims 6.

### Cell uptake and internalization studies

HSC-3 cells were seeded into 3.5 cm^2^ culture dish at a density of 2 × 10^6^, next cells were incubated with AF750-6Ahx-Sta-BBN or NGO-AF750-BBN at 37 °C for 15, 30, 60 and 120 min, respectively. Then, cells were washed three times with chilled PBS and harvested for flow cytometry measurements. Experiments were performed three times. For internalization studies, HSC-3 cells were cultured in 24-well plates at 2 × 10^4^ cells/well and grown for 24 h. Next, the original medium was replaced with fresh medium containing 50 nM AF750-6Ahx-Sta-BBN or NGO-BBN-AF750. The cells were then incubated for 4 h. After washing the cells three times with PBS, 2.5% paraformaldehyde was used to fix the cells. Then DAPI (Excitation, 488 nm) was used to stain the nuclei of the cells. Finally, the cells were visualized under OLYMPUS FV1200 confocal laser scanning microscope (Olympus, Osaka, Japan). In addition, HOK cells were used as a GRPR-negative control group and cultured with NGO-BBN-AF750, the above experiment was performed and compared to the HSC-3 cells.

### In vitro cellular toxicity

CCK8 cell viability assays were performed in HSC-3 cells to evaluate the effects of NGO-BBN-AF750 on cell toxicity. Briefly, HSC-3 cells were seeded in 96-well plates at a density of 5,000 per well and incubated for 24 h. The original medium was replaced with fresh medium, and added 0.05 nM NGO-BBN-AF750 or PBS (control group). After incubated for another 24 h, the medium was replaced with 90 µL fresh medium containing 10 µL CCK-8 for 30 min. And the absorbance was measured at 450 nm on ThermoFisher automated cell counter (ThermoFisher Scientific, USA).
